# Keratins Are Altered in Intestinal Disease-Related Stress Responses

**DOI:** 10.3390/cells5030035

**Published:** 2016-09-10

**Authors:** Terhi O. Helenius, Cecilia A. Antman, Muhammad Nadeem Asghar, Joel H. Nyström, Diana M. Toivola

**Affiliations:** 1 Faculty of Science and Engineering, Cell Biology/Biosciences, Åbo Akademi University, Tykistökatu 6A, 20520 Turku, Finland; terhi.helenius@abo.fi (T.O.H.); cecilia.antman@abo.fi (C.A.A.); masghar@abo.fi (M.N.A.); joel.nystrom@abo.fi (J.H.N.); 2 Turku Center for Disease Modeling, University of Turku, 20520 Turku, Finland

**Keywords:** keratin, stress, recovery, inflammation, antibiotics, aging, lipopolysaccharide, colitis, phosphorylation, acute, chronic

## Abstract

Keratin (K) intermediate filaments can be divided into type I/type II proteins, which form obligate heteropolymers. Epithelial cells express type I-type II keratin pairs, and K7, K8 (type II) and K18, K19 and K20 (type I) are the primary keratins found in the single-layered intestinal epithelium. Keratins are upregulated during stress in liver, pancreas, lung, kidney and skin, however, little is known about their dynamics in the intestinal stress response. Here, keratin mRNA, protein and phosphorylation levels were studied in response to murine colonic stresses modeling human conditions, and in colorectal cancer HT29 cells. Dextran sulphate sodium (DSS)-colitis was used as a model for intestinal inflammatory stress, which elicited a strong upregulation and widened crypt distribution of K7 and K20. K8 levels were slightly downregulated in acute DSS, while stress-responsive K8 serine-74 phosphorylation (K8 pS74) was increased. By eliminating colonic microflora using antibiotics, K8 pS74 in proliferating cells was significantly increased, together with an upregulation of K8 and K19. In the aging mouse colon, most colonic keratins were upregulated. In vitro, K8, K19 and K8 pS74 levels were increased in response to lipopolysaccharide (LPS)-induced inflammation in HT29 cells. In conclusion, intestinal keratins are differentially and dynamically upregulated and post-translationally modified during stress and recovery.

## 1. Introduction

Keratin (K) intermediate filaments (IF) are cytoskeletal proteins that protect the cell from mechanical and non-mechanical stress [1]. The most common simple epithelial keratins (SEK) are K7, K8, K18, K19 and K20, and they are divided into acidic type I (K18-K20) and basic type II (K7-K8) keratins. Type I and type II keratins form non-covalent obligate heteropolymers in a 1:1 ratio, and they are expressed in specific pairs and in a tissue-specific manner [2]. For example, only K8 and K18 are expressed in hepatocytes, whereas all SEKs are found in intestinal epithelia [3]. Keratins are regulated by posttranslational modifications (PTM), and phosphorylation is the most common and best described PTM [4]. For instance, Serine 74 (S74) is the major amino acid on K8 that is phosphorylated during stress, apoptosis and mitosis [4]. Unlike in skin and liver, where keratin mutations are known to cause and predispose to disease, respectively [5,6,7,8], genetic studies have been unable to show a convincing link between keratin mutations and IBD pathogenesis [9,10,11], even if a few IBD patients with keratin mutations have been described [11] and the *KRT8* gene is located within the IBD2 locus on chromosome 12 [12]. K8 mutations could therefore be predisposing factors for IBD [13,14]. In SEK transgenic mutant or knockout mice, a variety of hepatic disorders are the most commonly described phenotypes [9]. Mice without K8 (K8^−/−^ mice) develop colitis, hyperproliferation of the colonic crypts and diarrhea, a phenotype that resembles human ulcerative colitis [15,16,17,18], suggesting that keratins may be important in intestinal homeostasis. In addition, K8^−/−^ mice are highly sensitive to colorectal cancer in two models [19].

Keratins are abundant proteins that are frequently identified as differentially expressed proteins similarly as other stress proteins, such as heat shock proteins (HSPs) [20]. HSPs are upregulated on both mRNA and protein levels upon stress [21]. IFs and keratins are similarly upregulated and modified in stress situations [9,22] and during recovery from stress, e.g., as seen in liver [23,24,25,26,27], pancreas [28,29], kidney [30], lung [31], and skin [32,33,34]. Contrary to increased hepatic K8 and K18 levels in human liver disease [23], colonic K8, K18 and K19 levels have recently been reported to decrease in human colon during inflammatory stress, as observed in ulcerative colitis [35]. Furthermore, K7, K8 and K20 are increased in human colitis-associated dysplasia and colorectal cancer compared to healthy controls [36,37,38,39].

Based on these studies, we hypothesized that keratins play a role in the colonic stress response in a similar way as in other organs and as other stress proteins. The aim was to characterize the colonic stress-responsive keratins and to provide an overall screen of keratin levels in the colon during disease-related stress and recovery. In vivo murine stress models used were acute or chronic experimental colitis (dextran sulphate sodium (DSS)-treatment), broad-spectrum antibiotics and high age). LPS-induced inflammation was used as an in vitro stress model.

## 2. Materials and Methods

### 2.1. Mice

Two to three month old FVB/n mice (chronic DSS-treatment and antibiotic-treatment), 2–2.5 month old Balb/c mice (acute DSS) and 14 month old FVB/n mice were housed at the Central Animal Laboratory of the University of Turku. Mice were treated according to the approved animal study protocol issued by The State Provincial Office of South Finland. Following treatment, mice were sacrificed by CO_2_ inhalation, the colon was excised and washed with phosphate buffer saline (PBS), and samples were collected in liquid nitrogen, Optimum cutting temperature compound (O.C.T. Compound; Sakura Finetek, AJ Alphen aan den Rijn, The Netherlands) and RNA later (Qiagen, Venlo, The Netherlands) for further analysis as outlined below.

### 2.2. Antibodies

Primary antibodies used for Western blotting and immunofluorescence staining were mouse anti-K7 (RCK-105; Progen, Heidelberg, Germany), rat anti-K8 and rat anti-K19 (Troma I and Troma III, respectively; Developmental Studies Hybridoma Bank, Iowa, IA, USA), rabbit anti-K8 (273) and rabbit anti-K18 (275; kind gifts from J.E. Eriksson), rabbit anti-K20 (It-Ks 20.10; Epitomics, Burlingame, CA, USA), rat anti-Hsc70 (Enzo Life sciences; Farmingdale, NY, USA), mouse anti-K8 pS74 (LJ4; kind gift from M.B. Omary), rabbit anti-Ki67 (Abcam, Cambridge, MA, USA), rat anti-HSF2 (Abcam) and rabbit anti-IκB-α (Santa Cruz Biotechnology; Dallas, TX, USA). Secondary antibodies used for Western blotting were HRP-conjugated anti-mouse (GE healthcare, Little Chalfont, UK), anti-rat (GE healthcare and Cell Signaling Technology, Danvers, MA, USA) and anti-rabbit (Cell Signaling Technology) IgG antibodies. Secondary antibodies used for immunofluorescence staining were Alexa 488/Alexa 546 anti-mouse, Alexa 488 anti-rat and Alexa 488 anti-rabbit antibodies (Invitrogen, Carlsbad, CA, USA). Nuclei were stained with DRAQ5 (Cell Signaling Technology).

### 2.3. DSS-Induced Colitis

2%–2.5% dextran sulfate sodium (DSS; 40,000 Da, TdB Consultancy AB, Uppsala, Sweden) was administered in autoclaved drinking water to 2–2.5-month-old Balb/c mice for 7–8 days with or without recovery (7 days) to achieve a model for acute colitis [40,41,42]. For mimicking chronic colitis, 2-month-old FVB/n mice were treated one week with 2.5% DSS, followed by a two-week recovery period after which this cycle was repeated once [43] and the animals were sacrificed on day 45. Control mice for each experiment were age- and sex-matched, and were treated equally as DSS-treated mice, except that they received autoclaved drinking water without DSS. A disease activity index (DAI) was used to determine disease severity. The DAI was calculated by grading body weight loss (1 point per each 5% of body weight loss), presence of blood in stool (0 = none; 1 = blood in stool pellets; 2 = clotted or fresh blood at anus) and stool consistency (0 = normal; 1 = formed but very soft; 2 = slightly loose; 3 = liquid).

### 2.4. Antibiotic-Treatment

Eighteen to nineteen days old FVB/n mice were treated with broad-spectrum antibiotics (vancomycin and imipenem at 68 mg/kg body weight/day each) in drinking water for 56 days before sacrifice [17]. Control mice were age- and sex-matched and were treated equally to mice treated with antibiotics, except that they received drinking water without antibiotics.

### 2.5. In Vitro LPS-Treatment of HT-29 Cells

HT-29 cells were grown to 80% confluency in RPMI-1640 medium supplemented with 10% FCS, 2 mM l-glutamine and 100 U/mL penicillin/streptomycin and treated with 500 ng/mL or 1000 ng/mL LPS (Sigma, Saint-Louis, MI, USA) in PBS. Control cells were treated with equal volumes of PBS. Cells were harvested 48 h after beginning of treatment and processed for immunoblotting and immunofluorescence staining.

### 2.6. Immunoblotting and Immunofluorescence Staining

Samples for protein analysis of distal and proximal colon (DSS-treated, antibiotic-treated old mice and control mice) were suspended in 24 μL homogenization buffer (0.187 M Tris-HCl pH 6.8, 3% SDS, 5 mM EDTA) per 1 mg of tissue and homogenized by 75 strokes in a Potter-Elvenhjelm tissue homogenizer in order to obtain total tissue lysates. Tissue lysates, and untreated or LPS-treated HT-29 cells collected in homogenization buffer (0.187 M Tris-HCl pH 6.8, 3% SDS, 5 mM EDTA) were incubated at 95 °C for 5 min and sheared with a 27G needle. Protein concentrations were measured with a Pierce BCA protein assay kit (Thermo scientific, Waltham, MA, USA), samples were normalized and 20 μg protein per sample was separated by SDS-PAGE, transferred to a polyvinylidene fluoride membrane (PVDF) and analyzed by immunoblotting. Protein bands were quantified with Image J software (National Institutes of Health) and normalized to loading control (Hsc70).

Fresh frozen colon samples in O.C.T. Compound were cryosectioned (6 μm), fixed in −20 °C acetone for 10 min and immunostained as described in [44]. HT-29 cells were grown on microscope cover glasses, treated with or without LPS, and fixed in −20 °C acetone for 10 min prior to immunofluorescence staining. Samples were analyzed with a Leica TCS SP5 matrix confocal microscope (Leica, Mannheim, Germany). Quantitative analysis of K8 pS74 (LPS), K20 (old mice) and Ki67-positive (antibiotics) cells was done by counting the percent of K8 pS74-positive cells/all cells or the number of K20 or Ki67-positive cells/crypt in 20 crypts. Mitotic cells in K8 pS74-staining on LPS-treated cells were determined by confocal analyses on DNA-staining by DRAQ5. In chronic DSS analysis, K20-positive crypt areas were measured and indicated as μm/total crypt length.

### 2.7. Gene Transcription Analysis

RNA was extracted from total colon lysate samples (acute and chronic DSS, antibiotic-treatment) collected in RNA later (Qiagen, Hilden, Germany) using a RNA isolation kit (Nucleospin RNA, Macherey-Nagel, Düren, Germany). After cDNA synthesis (Promega, Madison, WI, USA), Taqman 7900HT assay (Applied Biosystems, Foster city, CA, USA) was done using β-actin as an endogenous control. The primers (Oligomer, Helsinki, Finland) and probes (Universal probe library, Roche, Basel, Swizerland) that were used are described elsewhere [45]. Fold change differences were analyzed with RQ Manager (Applied Biosystems, Foster city, CA, USA) and Microsoft Excel (Microsoft, Redmond, WA, USA).

### 2.8. Statistical Analysis

Statistical calculations were performed using Microsoft Excel and GraphPad PRISM. Student’s t-test was used for statistical analysis. Quantitative data is shown as average ± error bars, which show standard deviation (SD).

## 3. Results

### 3.1. Murine DSS Colitis in the Acute Phase and in the Recovery Phase Display Altered Keratin Expression and Increased K8 pS74 Levels

**Figure 1:**
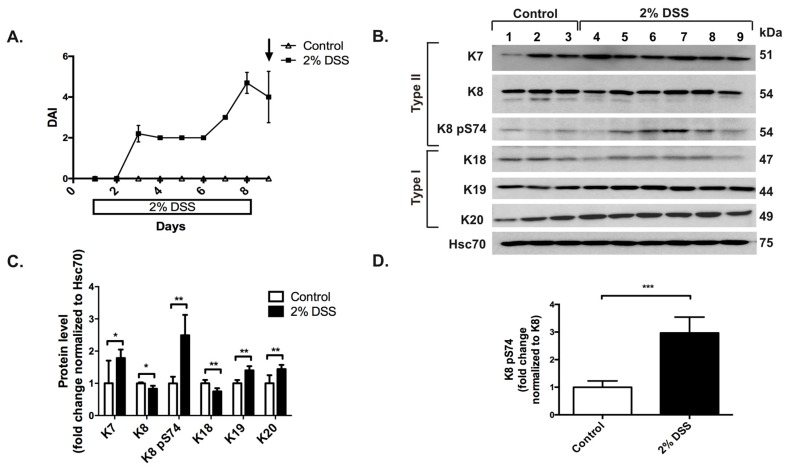

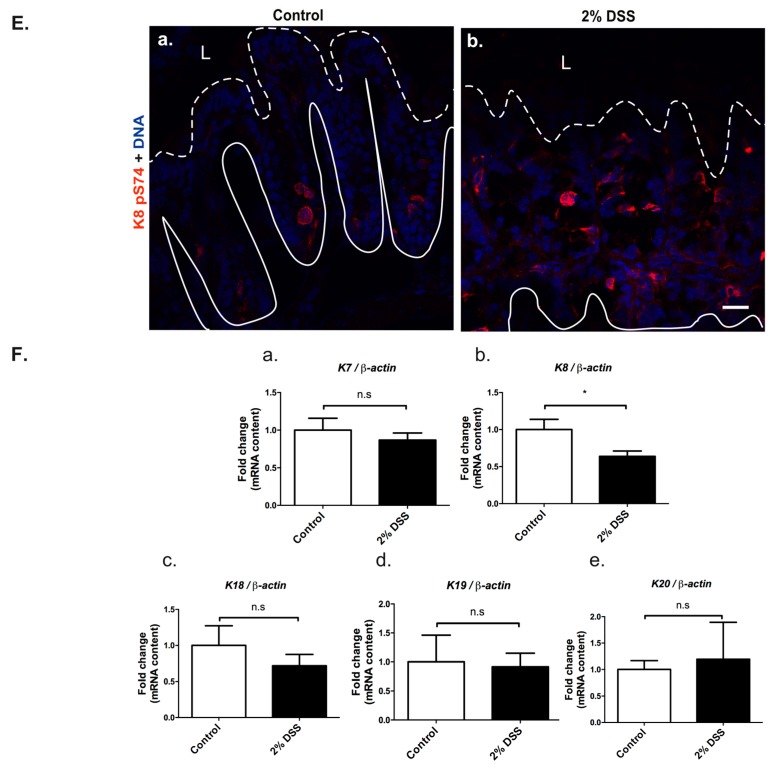
Experimental 2% dextran sulphate sodium (DSS)-induced colitis alters keratin levels and increases K8 phosphorylation at S74. To induce active murine colitis, mice (*n* = 3 for controls, lanes 1–3 in B and *n* = 6 for DSS-treated, lanes 4–9 in B; strain Balb/c) were treated with 2% DSS in drinking water. Samples were collected for protein, RNA and immunofluorescence analysis 8 days after the start of the treatment. (**A**) The daily DAI was calculated on the basis of measurements of body weight loss, stool consistency and presence of blood in stool in order to quantify colitis progression. The arrow indicates time point of sacrifice and sample collection. (**B**,**C**) Downregulation of K8 and K18 and upregulation of K7, K19 and K20 was detected and quantified using Western blot analysis of colon protein samples normalized by protein assay and the loading control Hsc70. * *p* < 0.05, ** *p* < 0.01. (**D**) Despite the K8 downregulation, an increase in K8 pS74 as normalized to K8 levels was seen. *** *p* < 0.001. (**E**) Immunofluorescence staining of K8 pS74 (red) showed more K8 pS74 positive cells in DSS-treated colons (panel b) compared to controls (panel a). Lines indicate the location and orientation of colonic crypts; dashed lines indicate the border between lumen and epithelium and solid lines the border between epithelium and basement membrane. Nuclei (DNA) are shown in blue. L = Lumen, scale bar = 50 μm. (**F**a–**F**e) Gene transcription analysis of K7, K18, K19 and K20 showed no significant changes, while K8 gene expression was decreased compared to control mice (normalization to β-actin). n.s = not significant, * *p* < 0.05. Numerical data are shown as average ± SD. Statistical significance for Western blot and mRNA data was determined by t-test.

In order to define if keratin levels are altered in an acute model of colitis, protein and mRNA levels for intestinal keratins were analyzed in samples collected from mice on day 8 after treatment with 2% DSS administered in drinking water. The DAI (Figure 1A), which was used to follow colitis progression and calculated on the basis of daily measurements of body weight loss, stool consistency and occult blood in stool (see Materials and Methods section), showed that the highest disease activity occurred on days 8–9 after the start of the experiment, and samples were collected on day 9. On protein level, K7, K19 and K20 were significantly upregulated while a minor downregulation of K8 and K18 was observed in the acute colitis-model compared to control mice (Figure 1B,C). The phosphorylation levels of the main stress-induced phosphorylation site K8 S74 were increased 2.5-fold, as seen by immunoblotting (Figure 1B,D). This was confirmed by confocal microscopy analysis where an increased number of K8 pS74-positive cells were seen higher up in the crypts after DSS-treatment compared to controls (Figure 1(Ea,b)).

Except for slightly lower levels of K8 gene expression (Figure 1(Fb)), no significant changes in mRNA levels for K7 (Figure 1(Fa)), K18 (Figure 1(Fc)), K19 (Figure 1(Fd)) or K20 (Figure 1(Fe)) were detected in the acute murine DSS-colitis samples.

**Figure 2:**
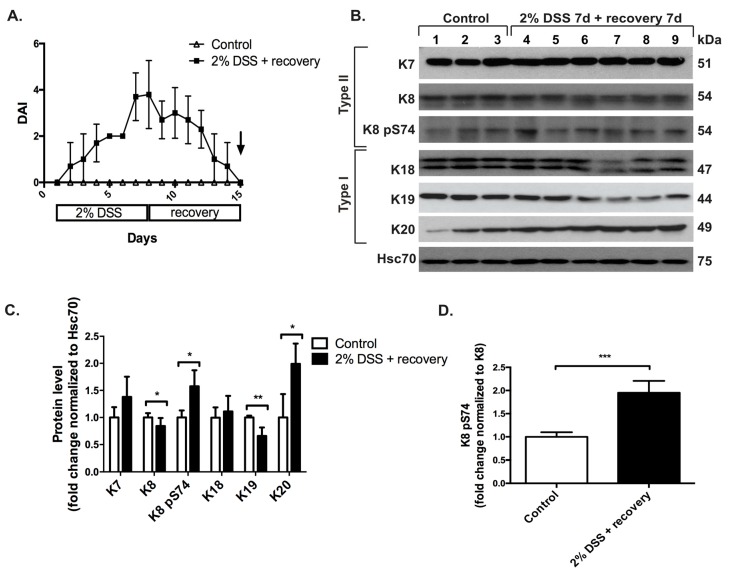
Acute DSS-associated keratin level alterations are not normalized after 7 days of recovery from DSS colitis even if the colon DAI is normalized. Mice (*n* = 3 for controls, lanes 1–3 in B, *n* = 6 for DSS, lanes 4–9 in B; strain Balb/c) were treated with 2% DSS for 7 days followed by 7 days of recovery time, after which samples were collected for protein and immunofluorescence analysis. (**A**) The daily DAI was calculated on the basis of body weight loss, stool consistency and presence of blood in stool during the whole experimental time of 14 days in order to monitor progression of colitis and recovery. The arrow indicates time point of sacrifice and sample collection. (**B**,**C**) A downregulation of the major keratin pair K8 and K19, together with upregulation of K20 and slight increase in K7, was seen when analyzed by Western blotting as described in Figure 1. * *p* < 0.05, ** *p* < 0.01. (**D**) K8 pS74 levels were normalized to K8 protein levels. Despite of the downregulation of K8, an increase in K8 pS74 was seen. *** *p* < 0.001. Numerical data are shown as average ± SD. Statistical significance for Western blot data was determined by t-test.

In order to investigate if keratin expression is also altered during the recovery phase after colitis-induced stress, induction of acute DSS-colitis with 2% DSS was followed by one week of normal water and recovery. The successful recovery from acute DSS-colitis was seen as the normalization of the DAI-index on day 15, 7 days into the recovery period (Figure 2A; the same day as when samples for protein analysis were collected). However, on the molecular level, keratin protein levels were not fully recovered, since a similar but not identical protein expression pattern was seen as in acute DSS-colitis without recovery (Figure 1B–D), where type II K8 was downregulated together with type I K18. Indeed, the addition of a one-week recovery time downregulated K19 instead of K18, whereas K7 (trend), and K20 were upregulated (Figure 2B,C). The K8 phosphorylation at S74 was still slightly elevated (Figure 2B,D), indicating that even if the physical parameters reflected by the DAI show recovery, the colonic epithelium has not yet recovered on molecular level as indicated by elevated stress-mediated keratin expression and phosphorylation levels.

### 3.2. The Type I-Type II Pair K20-K7 Is Upregulated after Chronic DSS-Colitis

**Figure 3:**
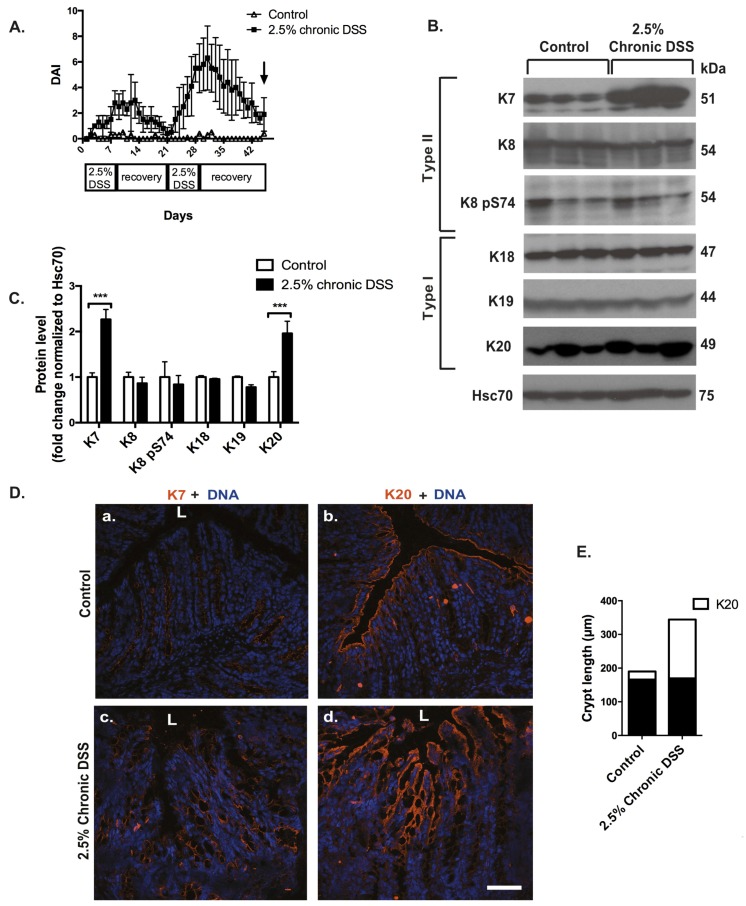

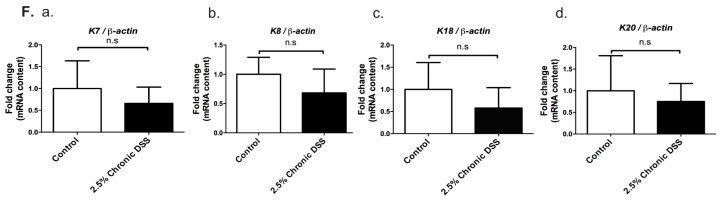
K7 and K20 are upregulated keratins in chronic DSS-colitis. To induce chronic colitis, mice (*n* = 3 for controls, *n* = 3 for DSS-treated; strain FVB/n) were given two one-week cycles of 2.5% DSS with two weeks of recovery with normal drinking water after each cycle. Samples for protein, RNA and immunofluorescence analysis were collected at the end of the second recovery period (A, arrow). (**A**) The daily DAI was calculated on the basis of body weight loss, stool consistency and presence of blood in stool during the whole experimental time of 45 days to follow colitis progression during DSS-treatment and the 2-week recovery periods. (**B**,**C**) A significant upregulation of K7 and K20 protein levels (as quantified in Figure 1) was seen after chronic DSS-treatment. *** *p* < 0.001. (**D**) Immunofluorescence staining shows that K7 (panels a, c in red) was expressed all over the crypt and also in the top-most crypt cells after chronic DSS-treatment (panel c) compared to controls (panel a). K20 (panels b, d in red) showed a broader crypt-distribution in response to chronic DSS (panel d) compared to controls (panel b). L = lumen, scale bar = 50 μm. Nuclei (DNA) are shown in blue. (**E**) Crypt length and K20-positive crypt areas (white bars) compared to K20 negative crypt areas (black bars) were quantified in controls and after chronic DSS-treatment. (**F**a–d) Gene transcription analysis from samples collected on day 45 as indicated in (**A**)) showed no significant changes in the gene transcription of K7, K8, K18 and K19. n.s = not significant. Numerical data are shown as average ± SD. Statistical significance for Western blot and mRNA data was determined by t-test.

To mimic the chronic and reoccurring IBD seen in humans, mice were subjected to a model of cyclic DSS-treatment consisting of two one-week cycles of 2.5% DSS with two weeks of recovery after each cycle. The cyclic nature of the DSS treatment and recovery was reflected by a three-fold increase in DAI in the first cycle of DSS, followed by a complete recovery of DAI at the end of the two-week recovery time. In the second cycle of DSS, a six-fold increase in DAI was observed, after which only a nearly complete recovery was gained at day 45 (17 days of recovery) (Figure 3A). At this time point, K7 and K20 protein levels were 1.5-fold upregulated (Figure 3B,C). K7, which is expressed in the lower and middle parts of the crypts under basal conditions (Figure 3(Da)), was distributed all over the crypts in response to chronic DSS-treatment (Figure 3(Dc)), verifying the increase in K7 seen by protein analysis (Figure 3B). K20, which is present only in the upper-most epithelial cells of the colonic crypts under basal conditions (Figure 3(Db)), showed an increased amount of K20-positive cells and an expanded distribution of K20 further down in crypts in response to chronic DSS-treatment (Figure 3(Dd)). The enlarged K20 zone coincided with the top-most part of the extended crypt as shown by the quantification of K20-positive areas in control and DSS-treated mice (Figure 3E). Interestingly, the distribution of the major colonic keratins, i.e., K18 and K19, was not altered (Supplementary Materials Figure S1) in response to chronic DSS-treatment.

Protein analysis of K8, K18 and K19 did not show any changes in response to chronic DSS-treatment compared to control mice (Figure 3B,C). Similar to acute DSS, no changes in the gene expression of colonic K7 (Figure 3(Fa)), K8 (Figure 3(Fb)), K18 (Figure 3(Fc)) or K20 (Figure 3(Fd)) were seen after chronic DSS.

### 3.3. Removal of Microbiota by Oral Broad-Spectrum Antibiotics-Treatment in Mice Causes an Upregulation of the Main Colonic Keratins K8 and K19 and an Increase in the Phosphorylation of K8 at S74

**Figure 4:**
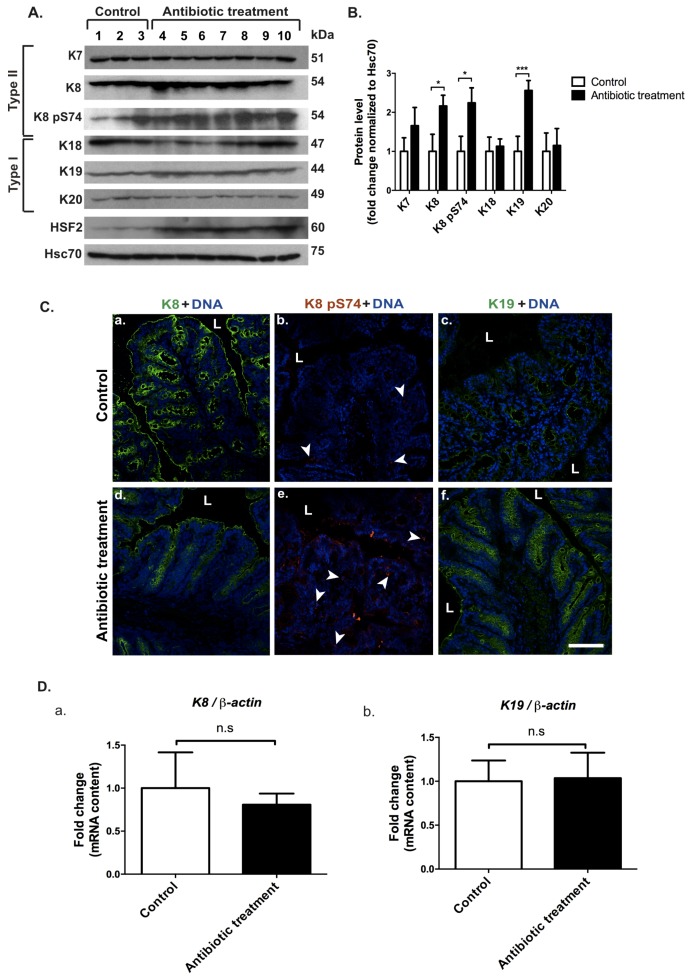
K8 and K19 are upregulated after the depletion of microbiota by oral antibiotic treatment. Mice (*n* = 3 for controls, lanes 1–3 in (**A**), *n* = 7 for antibiotic-treated, lanes 4–10 in (A); strain FVB/n) were treated with or without oral broad-spectrum antibiotics for 56 days and samples for protein, RNA and immunofluorescence analysis were collected at the end of the treatment. The samples were then analyzed to assess changes in keratin levels in response to the depletion of microbiota. (**A**,**B**) The levels of the main colonic keratins K8 and K19 were upregulated after antibiotic-treatment, along with increased levels of stress-responsive HSF2 and K8 pS74 when quantified as in Figure 1 and shown as average ± SD. * *p <* 0.05, *** *p <* 0.001. (**C**) The increase of K8 (panel a, d in green), K19 (panel d, f in green) and K8 pS74 (panel b, e in red arrow heads) after antibiotic-treatment was confirmed by confocal microscopy. Nuclei (DNA) are shown in blue. L = lumen, scale bar = 50 μm. (**D**a,b) Gene transcription analysis of K8 (**D**a) and K19 (**D**b) normalized to β-actin showed no significant changes. n.s = not significant. Numerical data are shown as average ± SD. Statistical significance for Western blot and mRNA data was determined by t-test.

Since the use of antibiotics in treatment of many human diseases can be considered a stress-factor for the colon due to the elimination of the health-promoting intestinal bacteria [46], mice were treated with broad-spectrum antibiotics for 8 weeks and analyzed for keratin levels. A significant 2.3-fold upregulation of the main intestinal keratins, K8 and K19, together with an increase in K8 pS74 was observed by Western blot (Figure 4A,B), and confirmed by immunofluorescence staining (Figure 4C). Co-immunostaining of K8 pS74 with the proliferation marker Ki67 revealed a slight increase in K8 pS74 in proliferating cells compared to non-proliferative cells following antibiotic-treatment (Supplementary Materials Figure S2). However, mice challenged with antibiotic-treatment showed no change in K8 (Figure 4(Da)) and K19 (Figure 4(Db)) gene expression. K7, K18 and K20 were unchanged regarding protein amounts and localization (Figure 4A,B, Supplementary Materials Figure S3). Interestingly, to confirm the activation of the colonic stress-response following the oral in vivo murine antibiotic-treatment, a strong upregulation of the stress-mediated heat shock factor 2 (HSF2) was seen (Figure 4A,B).

### 3.4. K8, K18 and K20 Are Upregulated in Proximal Colon and K7 and K19 in Distal Colon from Old Mice

**Figure 5:**
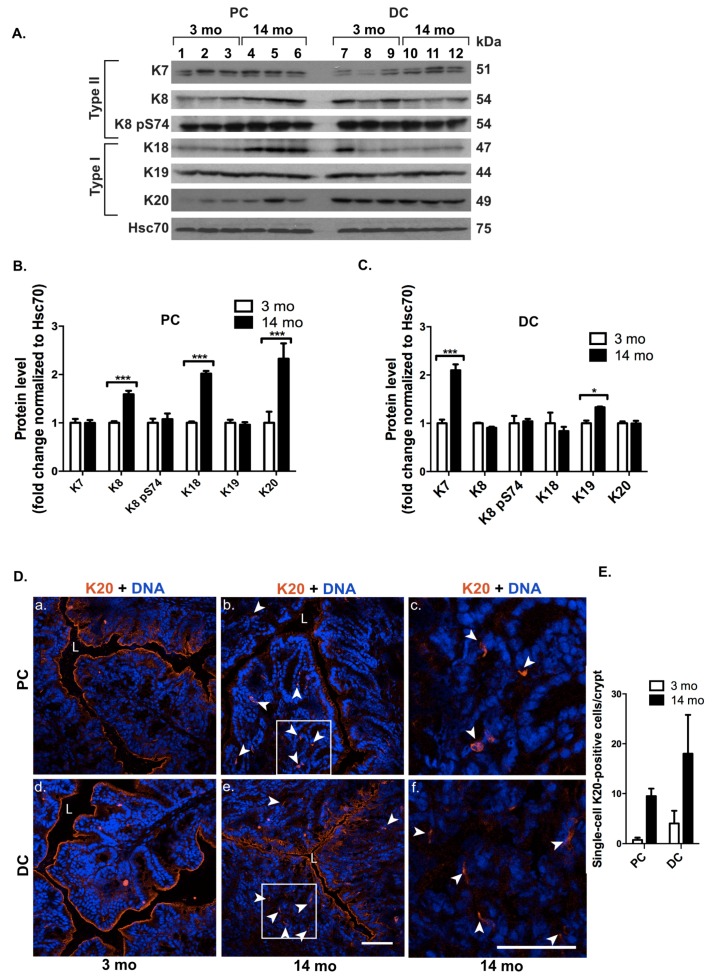
Ageing leads to upregulation of all colonic keratins in DC and PC in a differential manner. Proximal colon (PC) and distal colon (DC) keratin levels and distribution were analyzed in 3 and 14 months (mo) old mice (*n* = 3 for 3 months old mice, lanes 1–3 and 7–9, *n* = 3 for 14 months old mice, lanes 4–5 and 10–12) by protein analysis and immunofluorescence staining. (**A**–**C**) Keratin Western blotting and quantification (normalized to loading control Hsc70) of colon lysates showed that K8, K18 and K20 were upregulated in PC, whereas K7 was highly and K19 slightly upregulated in DC. * *p <* 0.05, *** *p <* 0.001. (**D**,**E**) The K20 upregulation in novel single cells below the luminal epithelium in 14 months old mice was confirmed by immunofluorescence staining in PC (panel a–b, b magnified from boxed area in c, arrow heads) and DC (panel d–e, e magnified from boxed area in f, arrow heads) as broader localization in the crypts. Nuclei (DNA) are shown in blue. L = lumen, scale bars = 50 μm. (**E**) The increased number of single-cell K20-positive cells/crypt in PC and DC of young and old mice is shown. *N* = 20 crypts. Numerical data are shown as average ± SD. Statistical significance for Western blot data was determined by t-test.

In order to analyze if colonic keratins are affected by ageing, keratin protein levels were analyzed in 3 and 14 month old mice. In PC, K8, K18 and K20 were highly upregulated in older mice compared to young adults (Figure 5A,B), while only K7 exhibited a high and K19 a minor upregulation in DC (Figure 5A,C). K20 upregulation was also confirmed by immunofluorescence staining (Figure 5D), showing more K20-positive cells lower down in the crypts in old mice compared to young mice (Figure 5E).

### 3.5. In Vitro LPS-Treatment Increases K8 and K19 Levels Together with K8 S74 Phosphorylation in HT-29 Cells

**Figure 6:**
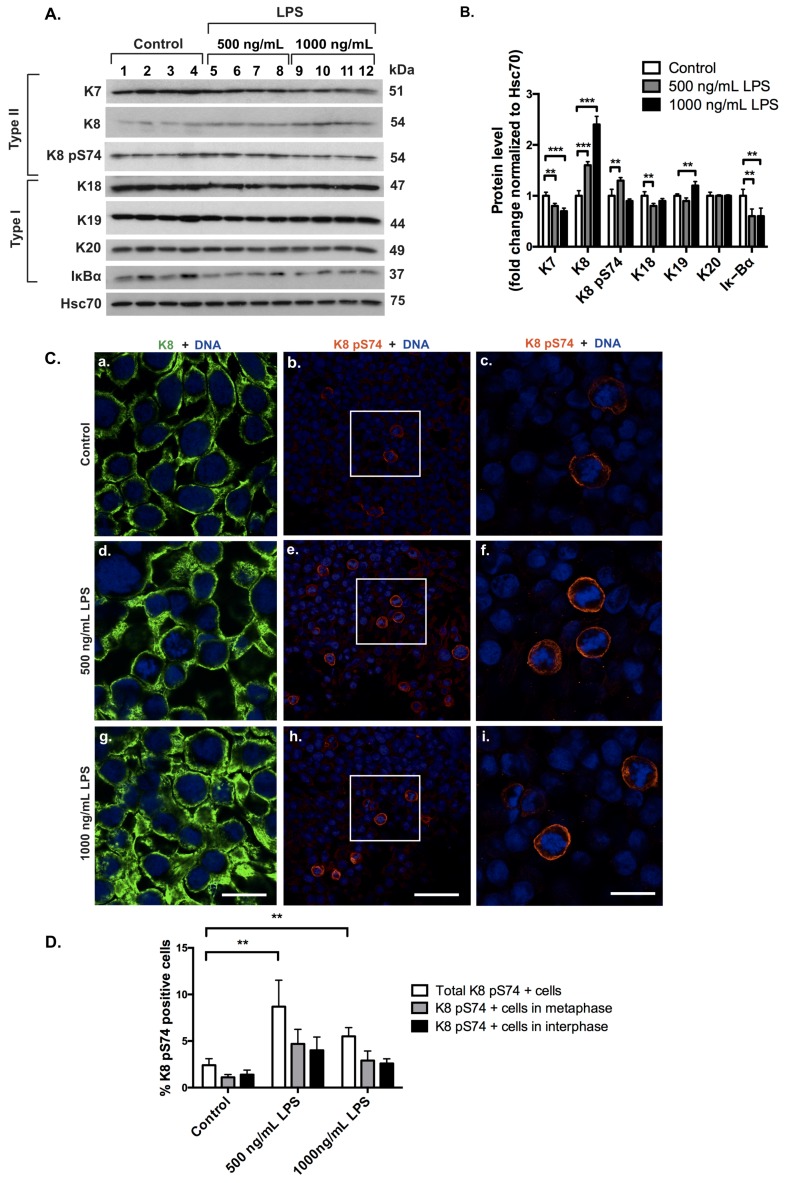
LPS, as a model for in vitro activation of inflammatory signaling pathways, upregulates K8 and K19 and increases phosphorylation of K8 at S74. Colorectal cancer HT-29 cells were treated with 500 ng/mL and 1000 ng/mL LPS for 48 h to induce inflammatory stress signaling. (**A**,**B**) LPS induced stress-activated inflammatory signaling, as seen by decreased levels of the NF-κB inhibitor IκBα, which was quantified by Western blotting and normalized to Hsc70. HT-29 cells showed an upregulation of the major colonic keratins K8 and K19 following treatment with 1000 ng/mL LPS. An increase in K8 pS74 was seen after treatment with 500 ng/mL LPS. ** *p* < 0.01, *** *p* < 0.001. (**C**) The upregulation of K8 (panels a, d and g; green) and the increase in K8 pS74 (panels b, e and h, and further magnified from white boxes to c, f and i, red) in response to LPS-treatment were confirmed by immunofluorescence staining. Nuclei (DNA) are shown in blue. Scale bar = 25 μm for a, c, d, f, g and i. Scale bar = 75 μm for b, e and h. (**D**) The increase in K8 pS74-positive cells was quantified, and showed a 6.3% increase in the amount of K8 pS74-positive cells after 500 ng/mL LPS-treatment compared to non-treated cells. For 1000 ng/mL LPS-treatment, the corresponding increase compared to non-treated cells was 3.1%. Numerical data are shown as average ± SD. Statistical significance for Western blot and immunofluorescence data was determined by t-test.

In order to study if keratin levels are affected in an in vitro cell culture inflammation model and to obtain a cell culture system for further mechanistic studies, we treated colorectal cancer HT-29 cells with 500 ng/mL and 1000 ng/mL of the bacterial LPS. In order to confirm that stress-activated inflammatory signaling is induced in LPS-treated HT-29 cells, the levels of IκBα, an inhibitor of NF-κB, were analyzed. The protein levels of IκBα were decreased after LPS-treatment, indicating that stress-activated inflammatory signaling was induced in this system (Figure 6A,B).

In contrast to the in vivo colitis model where the major keratins were downregulated, HT-29 cells treated with 1000 ng/mL LPS exhibited upregulated K8 and K19 protein levels 48 h after LPS-treatment, as shown by Western blot (Figure 6A,B) and confocal analyses (Figure 6C). A slight downregulation of K7 and K18 was seen in LPS-treated cells compared to control cells (Figure 6A,B), and an increase in K8 pS74 was seen in response to 500 ng/mL but not 1000 ng/mL LPS-treatment (Figure 6A–C). The increase in K8 pS74-positive cells was quantified, showing a 6.3% increase in the amount of K8 pS74-positive cells after 500 ng/mL LPS treatment compared to non-treated cells. The increase in K8 pS74-positive cells after 1000 ng/mL LPS-treatment compared to non-treated cells was 3.1%. Approximately half of the K8 pS74-positive cells were mitotic cells (Figure 6D). K20 protein levels and localization were not altered (Figure 6A,B, Supplementary Materials Figure S4). Therefore, this in vitro inflammation model confirms our hypothesis that keratins are stress-responsive proteins in the colon, and protect against stress through stress-mediated phosphorylation.

## 4. Discussion

In this study, we report that colonic keratins are upregulated during multiple murine models of human colonic stress and disease. We find that K7 and K20 are stress-responsive keratin upregulated in the three colitis regimen used, while the main colonic keratins K8 and K19 are increased in response to oral antibiotic-treatment. The stress-responsive pattern of K8/K19 is also seen in vitro in HT-29 cells after LPS-treatment. Most colonic keratins are upregulated in aging mice compared to young adult controls. Furthermore, phosphorylation of K8 at S74 is significantly increased during several of the stress-situations and in vitro.

During chronic colitis, increased protein levels and a wider crypt distribution of type II K7 (normally localized at the crypt-base and mid-crypt regions and type I K20 (normally localized in the crypt-top region [47]) is seen as the major differential keratin response in this model of chronic inflammation. Expression of keratins in a different cell type, tissue or cell compartment has previously been reported for keratins in stress situations in simple epithelia and in skin [22]. For example, K23 was recently suggested, together with K7 and K19, to be a novel stress-inducible ductal marker in liver disease [27]. Stress-induced keratin-expression has also been shown in response to disease-related stress in kidneys, where all renal keratins of the collecting ducts were relocalized to a more cytoplasmic pattern upon stress [30]. In the exocrine pancreas, K19 and K20 are upregulated and redistributed together with an increase in cytoplasmic filaments in response to pancreatitis, compared to an apical and lateral distribution under normal conditions [28]. Even if a 1.5–2.5-fold increase in specific keratins is seen in most colonic stress-conditions tested here, a slight decrease in K8, K18 and K19 can be observed in the acute DSS-models. A recent human study reports similar decreased K8, K18 and K19 levels in ulcerative colitis patients [35], and such downregulation could be explained by a local destruction of the single-layered colonic epithelium that can occur after acute DSS-treatment or in active human inflammatory disease.

Contrary to the upregulation of K7 and K20 in the inflammatory-stress model, the removal of colonic microbiota upregulated another set of stress-responsive keratins beyond normal levels, the main intestinal keratins type II K8 and type I K19, which during stress and basally are expressed throughout the crypt. Antibiotic treatment can cause mild gut inflammation, enlarged cecum, fewer Peyer’s patches in the small intestine and increased expression of several TLRs [46,48]. Antibiotic therapy is also known to prevent and treat genetic colitis models such as K8^−/−^ [16] and interleukin (IL)-10^−/−^ [49] mouse models, which both display a spontaneous colitis phenotype. The activation of a stress-induced reaction following antibiotic treatment was shown by an increase in HSF2. Interestingly, a similar stress-induced increase in HSF2 expression has also been reported in human ulcerative colitis patients [50].

Ageing of the colon is associated with hyperproliferation [51,52,53] and age-mediated telomere shortening, which may lead to the development of colonic inflammation and epithelial damage [54]. The levels of apoptosis are simultaneously decreased in the aging colonic epithelium [53]. Here, we show that several intestinal keratins are robustly upregulated in older mice, mainly K8, K18 and K20 in PC and K7 and K19 in DC. Earlier studies have found that ageing is associated with an increase in luminal cells expressing K14 in primary cell cultures from the mammary gland [55]. Furthermore, the expression of K19 was increased in the lung inflammatory response in ageing mice [56]. Although the field lacks more similar studies about keratin expression in ageing, studies of other IF proteins in ageing have been reported. Glial fibrillary acidic protein (GFAP) is upregulated in the oxidative stress reaction in response to brain injury [57], vimentin is three-fold upregulated in senescent fibroblasts [58] and lamin A is upregulated and accumulated in premature cellular senescence [59].

Hepatic K8 is hyperphosphorylated in response to inflammation, leading to changes in the solubility of keratins and keratin-binding proteins [4,60]. Our results show that intestinal K8 phosphorylation at S74 is in a similar way significantly increased after in vivo acute DSS colitis, antibiotic-treatment and in vitro LPS-stress. As in human liver disease [61] and in kidney [30] and lung [62] disease models, where keratin hyperphosphorylation is a marker of stress and K8 phosphorylation protects the described organ from damage [63], we propose that the hyperphosphorylation of K8 could be used as such as a colonic stress marker.

No major changes in keratin mRNA levels were observed in any of the stress models studied here. Since the overall rate of mRNA-protein turnover is rapid in the colonic epithelium which requires constant renewal, and the time point when protein changes can be detected unlikely overlaps with the peak time point for mRNA levels, the situation in the colon is somewhat different than in liver [23], kidney [30] and exocrine pancreas [28] where keratin overexpression in response to disease-related stress has been detected on both protein and mRNA levels. In the CCl_4_ liver fibrosis model, a similar phenotype of unchanged mRNA levels for K8 was, however, seen in response to a chronic ongoing liver inflammation [25]. Although keratin mRNA and protein expression in diseased murine kidneys show an increase in keratin levels, the increase in protein levels are still not proportional to the mRNA-levels, suggesting an involvement of translational regulation of keratin expression [30]. K8 and K18 are also known to be degraded due to stress-mediated ubiquitination by the proteasome, as seen in human lung cells [64,65]. Overall, many studies have reported that mRNA and protein levels do not always correlate [66] and qualitative analyses of protein expression from mRNA transcripts are known to be insufficient [67] due to post-transcriptional mechanisms, different in vivo half-lives of proteins and errors in both protein and mRNA expression experiments [68]. We thus cannot rule out that the observed changes in keratin levels could be due to factors such as protein degradation and protein stability, which affect the overall protein levels. Further studies regarding protein stability should be addressed, for example by a time-dependent DSS-experiment, where the affected signaling pathways could be studied in more detail and any initial increases in mRNA levels detected. Other ex vivo models, such as colonic organoids treated with stressors (e.g., DSS, LPS) or protein stabilizers (MG132 and cycloheximide) could be helpful in analyzing the underlying mechanism responsible for keratin function in intestinal stress protection, which needs to be addressed next. The keratin transcription factors are still rather unknown and need to be further scrutinized during stress-conditions, although p53, sp1 and AP1 have been shown in the regulation of K8 and K18 transcription [69]. 

We also investigated whether keratin-levels are responsive to stress in a cell culture setting. When colorectal cancer HT-29 cells are challenged with bacterial LPS as a model of in vitro inflammation, K8 and K19 are increased. Under normal conditions, the intestinal microflora consists of a balance of gram-negative and gram-positive bacteria, shifted slightly to the dominance of gram-positive bacteria [70]. LPS is a major constituent of the gram-negative bacteria, which are predominant during intestinal inflammatory disorders such as IBD [71,72,73]. Therefore, during colitis, the intestinal lumen serves as a reservoir of LPS [74]. By treating HT-29 cells with LPS, the inflammatory TLR4 signaling pathway is activated [75] as shown in our study by the downregulation of IκBα, the inhibitor of NF-κB. The inflammatory signaling is achieved by the activation of caspase-7 and caspase-1 [76] and previous studies have confirmed that LPS-treatment does not affect the proliferation of HT-29 cells [77]. Supportive of our study is that treatment with *Bifidobacterium breve* induced an upregulation of K8 in HT-29 cells [78]. Interestingly, IL-6 treatment of Caco-2 cells induces K8 and K18 on protein and mRNA level and increases K8 phosphorylation, which is needed for the maintenance of the intestinal barrier often disrupted in intestinal stress [79]. In vitro colonocyte model systems may thus be useful for further studies to unravel the molecular mechanisms and dynamics of keratin induction during stress. 

To date, the occurrence of adolescent IBD is increasing, with the highest annual incidence of 24.3 cases per 100,000 persons reported in Europe [80]. Emerging results from many other tissue models demonstrate the protective role of keratins in disease-related stress situations. To this end, K8^−/−^ mice develop spontaneous colitis, while K8 heterozygous knockout mice (K8^+/−^) mice, with only 50% of normal colonic keratin levels, are more sensitive to DSS-induced colitis compared to wild-type mice [45]. Intestinal keratins are differentially and dynamically upregulated and post-translationally modified upon various sources of stress during colonic stress and recovery.

## 5. Conclusions

**Figure 7:**
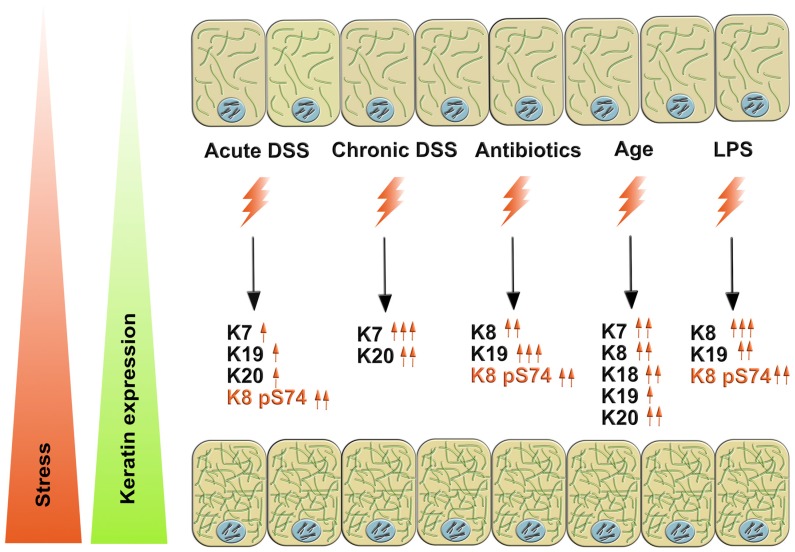
Intestinal keratins K7, K8, K18, K19 and K20 are stress-responsive proteins, which display a pair-wise and context-specific upregulation in response to stress. During acute and chronic DSS-stress, the K7–K20 pair is the predominantly overexpressed keratin pair. During murine antibiotic-treatment and in vitro LPS-stress, the keratin pair K8–K19 is overexpressed. During aging, all colonic keratins are upregulated, but most robustly K7, K8, K18 and K20.

The intestinal keratins K7, K8, K18, K19 and K20 can be considered as stress-responsive proteins, since they display a pair-wise acute and prolonged upregulation in response to different disease-related stresses and in recovery from stress (Figure 7). Surprisingly, the pair-wise keratin expression varies according to different stress-situations, with the predominant stress-responsive pair in DSS-stress being type II K7 and type I K20, while during antibiotic stress the main intestinal keratins, K8 and K19, are upregulated. The keratin stress response is predominantly seen at protein level, likely reflecting the fast renewal rate of the epithelial colonocytes and shorter duration of increased mRNA expression. Colorectal cancer cells in culture upregulate keratins and increase keratin phosphorylation upon LPS exposure, and they could thus be used to unravel the molecular mechanisms of keratins in stress protection.

## Supplementary Materials

The following are available online at www.mdpi.com/2073-4409/5/3/35/s1, Figure S1: K18 and K19 distribution is not altered in response to chronic DSS-treatment, Figure S2: A slight increase in K8 pS74 was seen in proliferating cells compared to non-proliferative cells following antibiotic treatment, Figure S3: The distribution of K18 and K20 is unaltered in response to antibiotic-treatment, Figure S4: The amount and distribution of K20 in LPS-treated HT-29 cells is unchanged.

## Abbreviations

The following abbreviations are used in this manuscript:

DAI	Disease activity index
DSS	dextran sulphate sodium
FCS	Fetal calf serum
HSF2	Heat shock factor 2
IF	Intermediate filament
IL	Interleukin
K	Keratin
K8^−/−^ mice	K8 knock out mice
L	Lumen
LPS	Lipopolysaccharide
O. C. T. Compound	Optimum cutting temperature compound
PBS	Phosphate buffered saline
PFA	Paraformaldehyde
PTM	Posttranslational modifications
PVDF	Polyvinylidene fluoride
pS74	Phosphorylated Serine 74
SD	Standard deviation
SEK	Simple epithelial keratin
