# Supplementary material for: Keratins Are Altered in Intestinal Disease-Related Stress Responses
Source: Cells. 2016 Sep 10;5(3):35. doi: 10.3390/cells5030035 (PMC5040977; doi:10.3390/cells5030035)
Supplement: Supplementary file 1 [file cells-05-00035-s001.pdf]

Supplementary Materials: Keratins Are Altered in Intestinal Disease-Related Stress Responses

Terhi O. Helenius, Cecilia A. Antman, Muhammad Nadeem Asghar, Joel H. Nyström and Diana M. Toivola

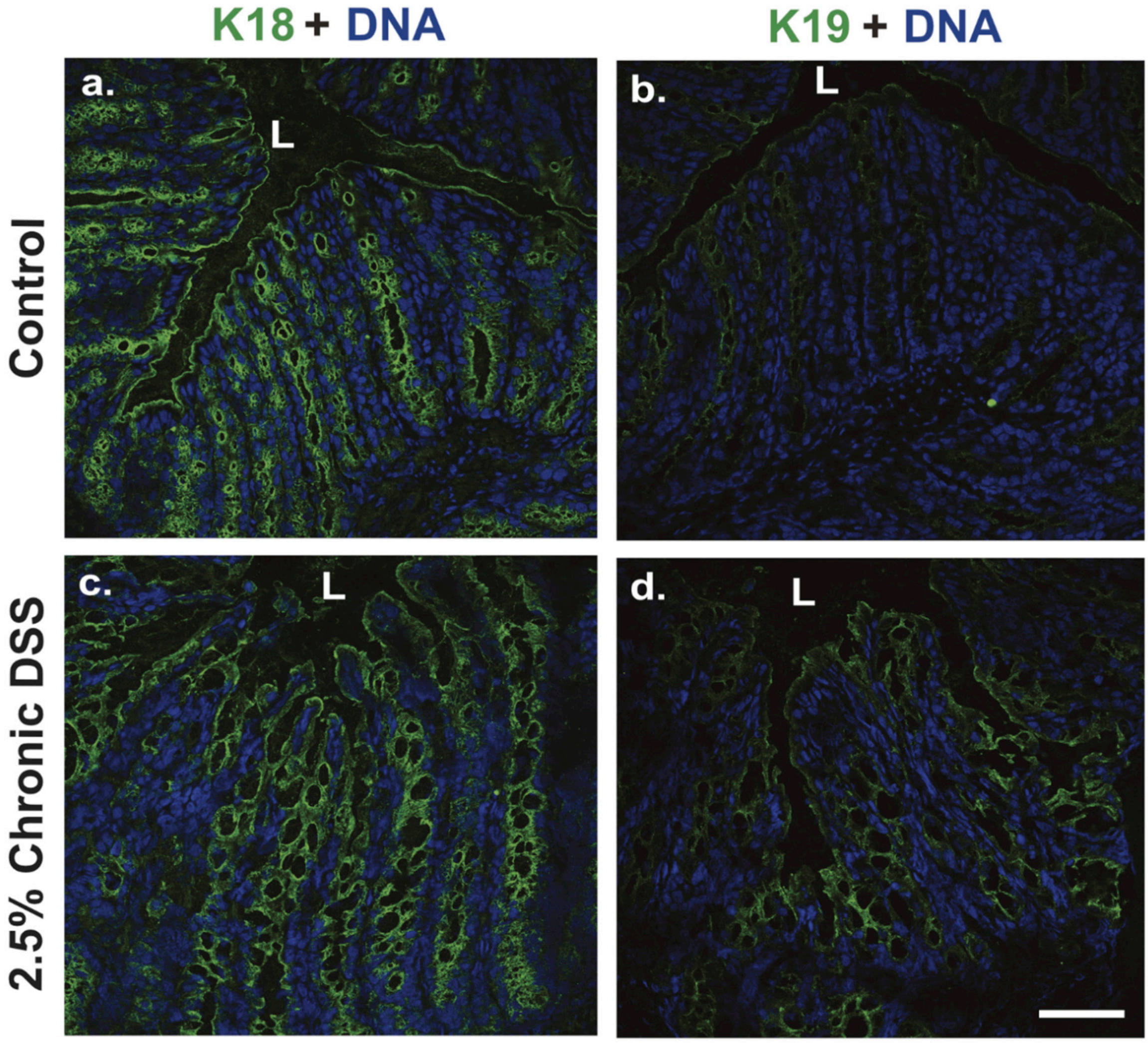

Figure S1. K18 and K19 distribution is not altered in response to chronic DSS-treatment. To induce chronic colitis, mice were given two one-week cycles of 2.5% DSS with two weeks of recovery with normal drinking water after each cycle. Tissue section samples were analyzed for K18 (**a**, **c** in green) and K19 (**b**, **d** in green) by immunofluorescence staining and confocal microscopy. Nuclei (DNA) are stained blue. Baseline crypt-base to -top distribution of K18 and K19 was not altered after 2.5% chronic DSS-treatment. L = Lumen, scale bar = 50 μm.

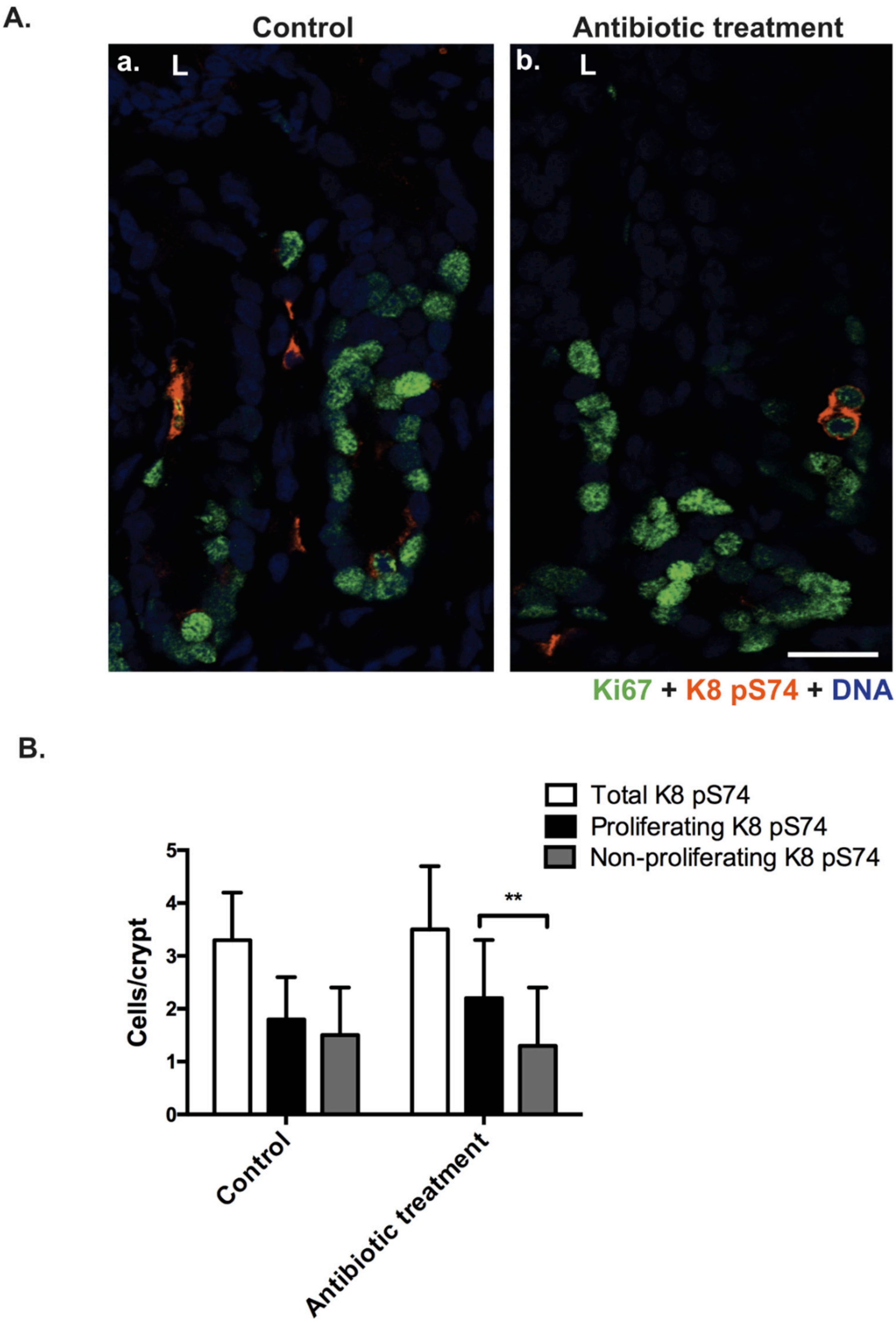

Figure S2. A slight increase in K8 pS74 was seen in proliferating cells compared to non-proliferative cells following antibiotic treatment. Control mice (Aa) were treated with oral broad-spectrum antibiotics (**A**b) for 56 days, samples for immunofluorescence analysis were collected at the end of the treatment, and (**A**) immunofluorescence staining of K8 pS74 (red) and the proliferation marker Ki67 (green) were analyzed. Proliferating (red and green cells) and non-proliferating K8 pS74-positive cells (red only cells) in the bottom of the crypts were imaged and quantified (**B**). The number of proliferating K8 pS74-positive cells/crypt (black bars) is increased compared to K8 pS74-positive non-dividing cells (gray bars). The total number of K8 pS74 cells is shown in white bars. The data is shown as averages ± SD and ** $p < 0.01$. Statistical significance was determined by t-test. Nuclei (DNA) are shown in blue. L = Lumen, scale bar = 50 μm

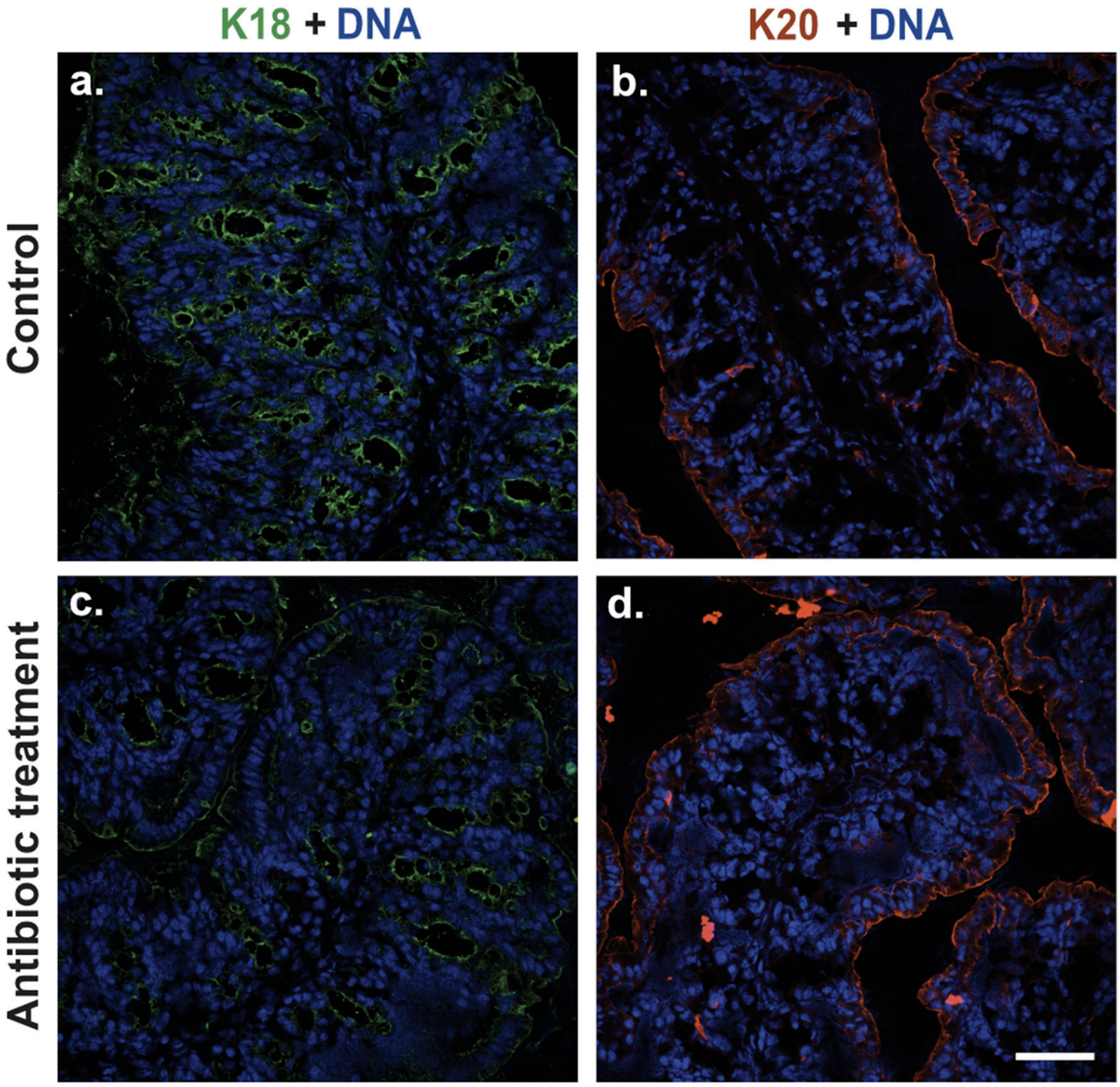

Figure S3. The distribution of K18 and K20 is unaltered in response to antibiotic treatment. Mice (n = 3 for controls, n = 4 for antibiotic-treated) were treated with oral broad-spectrum antibiotics for 56 days. Samples for immunofluorescence analysis were collected at the end of the treatment, and keratin distribution was analyzed in response to the depletion of microbiota. (**a**,**b**) The wide distribution of K18 (green) is unchanged in response to antibiotic treatment. (**c**,**d**) K20 (red) is found at the top of the crypts both under baseline conditions and after antibiotic treatment. Nuclei (DNA) are shown in blue. L = Lumen, scale bar = 50 μm.

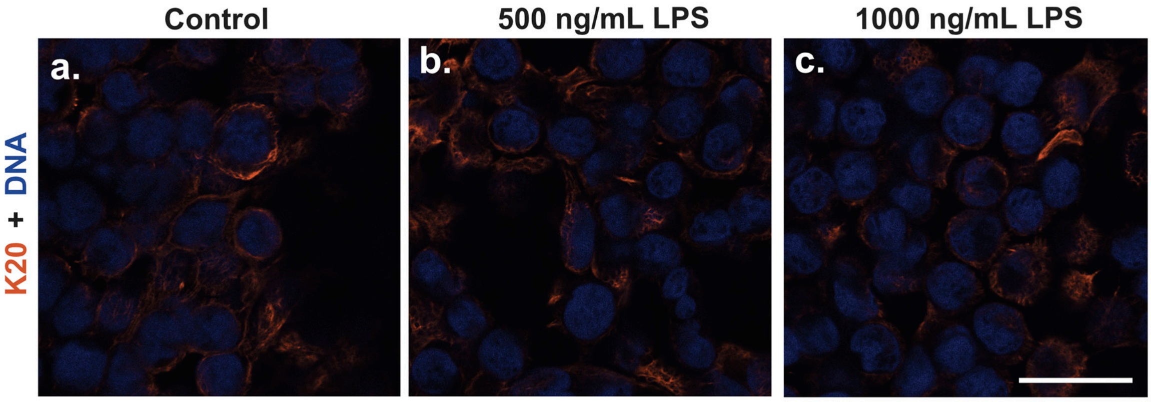

Figure S4. The amount and distribution of K20 in LPS-treated HT-29 cells is unchanged. Colorectal cancer HT-29 cells were treated with 500 ng/mL or 1000 ng/mL LPS for 48 h to induce inflammatory stress signaling prior to sample collection. The levels and distribution of K20 assessed by immunofluorescence staining and confocal microscopy in LPS-treated HT-29 cells (**b**,**c**) were unchanged compared to control cells (**a**). Nuclei (DNA) are shown in blue. Scale bar = 25 μm.
